# *KLOTHO* KL‐VS heterozygosity is associated with diminished age‐related neuroinflammation, neurodegeneration, and synaptic dysfunction in older cognitively unimpaired adults

**DOI:** 10.1002/alz.13912

**Published:** 2024-06-21

**Authors:** Ira Frahmand Driscoll, Sarah Lose, Yue Ma, Barbara B. Bendlin, Catherine Gallagher, Sterling C. Johnson, Sanjay Asthana, Bruce Hermann, Mark A. Sager, Kaj Blennow, Henrik Zetterberg, Cynthia Carlsson, Gwendlyn Kollmorgen, Clara Quijano‐Rubio, Dena Dubal, Ozioma C. Okonkwo

**Affiliations:** ^1^ Wisconsin Alzheimer's Disease Research Center and Department of Geriatrics University of Wisconsin School of Medicine and Public Health Madison Wisconsin USA; ^2^ Wisconsin Alzheimer's Institute Madison Wisconsin USA; ^3^ Geriatric Research Education and Clinical Center William S. Middleton VA Hospital Madison Wisconsin USA; ^4^ Department of Neurology University of Wisconsin School of Medicine and Public Health Madison Wisconsin USA; ^5^ Department of Psychiatry and Neurochemistry Institute of Neuroscience and Physiology Sahlgrenska Academy at the University of Gothenburg Mölndal Sweden; ^6^ Clinical Neurochemistry Laboratory Sahlgrenska University Hospital Göteborg Sweden; ^7^ Paris Brain Institute ICM Pitié‐Salpêtrière Hospital Sorbonne University Paris France; ^8^ Neurodegenerative Disorder Research Center Division of Life Sciences and Medicine and Department of Neurology Institute on Aging and Brain Disorders University of Science and Technology of China and First Affiliated Hospital of USTC Hefei PR China; ^9^ Department of Neurodegenerative Disease UCL Institute of Neurology, Queen Square London UK; ^10^ UK Dementia Research Institute at UCL London UK; ^11^ Hong Kong Center for Neurodegenerative Diseases Clear Water Bay Hong Kong PR China; ^12^ Roche Diagnostics GmbH Penzberg Germany; ^13^ Roche Diagnostics International Ltd Rotkreuz Switzerland; ^14^ Department of Neurology and Weill Institute for Neurosciences University of California San Francisco San Francisco California USA

**Keywords:** α‐synuclein, Alzheimer's disease, chitinase‐3‐like protein 1, glial fibrillary acidic protein, interleukin 6, neurogranin, protective factors, resilience, risk, S100 calcium‐binding protein B, triggering receptor expressed on myeloid cells

## Abstract

**INTRODUCTION:**

We examined whether the aging suppressor *KLOTHO* gene's functionally advantageous KL‐VS variant (KL‐VS heterozygosity [KL‐VS_HET_]) confers resilience against deleterious effects of aging indexed by cerebrospinal fluid (CSF) biomarkers of neuroinflammation (interleukin‐6 [IL‐6], S100 calcium‐binding protein B [S100B], triggering receptor expressed on myeloid cells [sTREM2], chitinase‐3‐like protein 1 [YKL‐40], glial fibrillary acidic protein [GFAP]), neurodegeneration (total α‐synuclein [α‐Syn], neurofilament light chain protein), and synaptic dysfunction (neurogranin [Ng]).

**METHODS:**

This Alzheimer disease risk‐enriched cohort consisted of 454 cognitively unimpaired adults (M_age _= 61.5 ± 7.75). Covariate‐adjusted multivariate regression examined relationships between age (mean‐split[age ≥ 62]) and CSF biomarkers (Roche/NeuroToolKit), and whether they differed between KL‐VS_HET_ (*N* = 122) and non‐carriers (KL‐VS_NC_; *N* = 332).

**RESULTS:**

Older age was associated with a poorer biomarker profile across all analytes (*P*s ≤ 0.03). In age‐stratified analyses, *KL‐VS_NC_* exhibited this same pattern (*P*s ≤ 0.05) which was not significant for IL‐6, S100B, Ng, and α‐Syn (*P*s ≥ 0.13) in KL‐VS_HET_. Although age‐related differences in GFAP, sTREM2, and YKL‐40 were evident for both groups (*P*s ≤ 0.01), the effect magnitude was markedly stronger for *KL‐VS_NC_*.

**DISCUSSION:**

Higher levels of neuroinflammation, neurodegeneration, and synaptic dysfunction in older adults were attenuated in KL‐VS_HET_.

**Highlights:**

- Older age was associated with poorer profiles across all cerebrospinal fluid biomarkers of neuroinflammation, neurodegeneration, and synaptic dysfunction.
- *KLOTHO KL‐VS* non‐carriers exhibit this same pattern, which is does not significantly differ between younger and older KL‐VS heterozygotes for interleukin‐6, S100 calcium‐binding protein B, neurogranin, and total α‐synuclein.
- Although age‐related differences in glial fibrillary acidic protein, triggering receptor expressed on myeloid cells, and chitinase‐3‐like protein 1 are evident for both KL‐VS groups, the magnitude of the effect is markedly stronger for KL‐VS non‐carriers.
- Higher levels of neuroinflammation, neurodegeneration, and synaptic dysfunction in older adults are attenuated in KL‐VS heterozygotes.

## 1 BACKGROUND

Age is the most potent risk factor for developing both Alzheimer's disease (AD) pathology and the clinical syndrome. Nonetheless, it has become evident that the relationship between aging and AD phenotypes is imperfect with ample evidence for interindividual heterogeneity.^1^, ^2^ This highlights the fact that successful aging in the absence of dementia is possible^3^, ^4^, ^5^, ^6^, ^7^, ^8^, ^9^, ^10^, ^11^, ^12^, ^13^, ^14^ and underscores the need for a shift in investigating not only the risk but also the protective factors against AD pathological changes with advancing age.

Klotho, an anti‐aging and longevity factor encoded by the *KLOTHO* gene, is a transmembrane protein and a circulating factor with a key role in cellular metabolism and body homeostasis.^15^, ^16^ Klotho plays a role in central nervous system maturation and aging, enhances synaptic and cognitive functions, and protects from neurodegeneration.^17^, ^18^, ^19^, ^20^, ^21^, ^22^ While the exact mechanisms of klotho action are under continued investigation, a few neuroprotective mechanisms have been suggested. For example, klotho may offer neuroprotection by modulating insulin‐like growth factor 1 signaling and oxidative stress,^22^ by affecting oligodendrocyte function and in turn preventing demyelination in the aging brain,^14^ or by enhancing synaptic function and counteracting adverse effects of pathologically elevated amyloid beta (Aβ) levels through enrichment of GluN2B‐containing N‐methyl‐D‐aspartic acid receptors.^18^

In humans, two *KLOTHO* single nucleotide polymorphisms (rs9536314 and rs9527025) segregate to form a functional haplotype KL‐VS, which modulates klotho secretion.^23^, ^24^, ^25^, ^26^, ^27^ Human empirical and meta‐analytic studies suggest that the functionally advantageous KL‐VS heterozygosity (KL‐VS_HET_) is associated with various favorable aging phenotypes, including longevity, better cardiovascular health, and better cognition.^18^, ^28^, ^29^, ^30^, ^31^ KL‐VS_HET_ is also associated with lesser AD neuropathology, namely, lower Aβ burden,^25^, ^32^, ^33^ and lower AD risk in apolipoprotein E (*APOE*) ε4 carriers.^33^ Moreover, our group recently reported that KL‐VS_HET_ attenuates adverse effects of age on memory and executive function^26^ and cerebrospinal fluid (CSF) tau both cross‐sectionally^26^ and longitudinally^27^ in a cognitively unimpaired cohort enhanced for AD risk.

Extracellular Aβ plaques and intraneuronal neurofibrillary tangles are the hallmark AD pathologies; thus, therapeutic strategies have largely been aimed at reducing them, with recent clinical trials showing some slowing of cognitive decline with anti‐amyloid therapies.^34^, ^35^, ^36^ However, plaque and tangle pathology only reflect part of the underlying disease process and highlight the need to explore additional pathophysiological perturbations associated with this multifactorial disease.^37^ Accordingly, tools for measuring several pathologies hold high potential, including several novel CSF biomarkers,^38^, ^39^, ^40^, ^41^ some of which are more extensively validated than others. The present study highlights several more established markers that tag the following processes: (1) neuroinflammation and glial activation (interleukin‐6 [IL‐6], S100 calcium‐binding protein B [S100B], triggering receptor expressed on myeloid cells [sTREM2], chitinase‐3‐like protein 1 [YKL‐40], and glial fibrillary acidic protein [GFAP]), (2) synaptic dysfunction (neurogranin [Ng]), and (3) neurodegeneration (total α‐synuclein [α‐Syn] and neurofilament light chain protein [NfL]), all of which change unfavorably with age or AD. Our objective is to examine whether KL‐VS_HET_ attenuates the deleterious effects of aging on these AD‐relevant processes and associated biomarkers in a cognitively unimpaired cohort enriched for AD risk.

## 2 METHODS

### 2.1 Participants

The current sample comprised 454 late‐middle‐aged and older, cognitively unimpaired adults from the Wisconsin Registry for Alzheimer's Prevention (WRAP),^42^ and the Wisconsin Alzheimer's Disease Research Center (WADRC) studies who were genotyped for *KLOTHO* and underwent CSF sampling. This cohort is enriched for AD risk based on both *APOE* ε4 allele (38%) and family history of AD (74%). Participants were considered cognitively unimpaired based on standardized, multidisciplinary, consensus diagnostic conferences that take into account functional impairments, medical/neurologic conditions that might impair cognition, and performance on a comprehensive battery of neuropsychological tests.^42^ A signed written consent was obtained from all participants prior to commencement of study procedures, which were approved by the University of Wisconsin Institutional Review Board.

### 2.2 Genotyping

A PUREGENE DNA Isolation Kit (Gentra Systems, Inc.) was used to extract DNA from blood. DNA concentrations were quantified via ultraviolet spectrophotometry (DU 530 Spectrophotometer, Beckman Coulter). Genotyping for *APOE* (rs429358 and rs7412) and *KLOTHO* (rs9536314 and rs9527025) was done by LGC Genomics using competitive allele‐specific polymerase chain reaction–based KASP genotyping assays. Quality control procedures have been previously published^25^, ^43^ and were deemed satisfactory. In our cohort, as expected based on HapMap and the literature,^25^, ^26^, ^27^ *KLOTHO* rs9536314 and rs9527025 were in perfect linkage disequilibrium.

**RESEARCH IN CONTEXT**

1. **Systematic review**: Literature on the role for the *KLOTHO KL‐VS* gene in central nervous system aging and Alzheimer's disease (AD) was reviewed. The functionally advantageous KL‐VS heterozygosity (KL‐VS_HET_) is associated with various favorable aging phenotypes, including better memory and executive function, lesser AD neuropathology, and lower AD risk. The present study examines whether the deleterious effects of aging on AD‐relevant processes of neuroinflammation, neurodegeneration, and synaptic dysfunction differ based on KL‐VS genotype in a cognitively unimpaired cohort enriched for AD risk.
2. **Interpretation**: Regardless of genotype, older age is associated with a poorer biomarker profile across all analytes. While this holds true for KL‐VS non‐carriers, the age‐related pattern is either not significant in KL‐VS heterozygotes, or if still significant, the magnitude of the effect is markedly less than that observed for the non‐carriers. KL‐VS_HET_ hence appears to protect against an array of deleterious age‐related biomolecular changes.
3. **Future directions**: While the mechanism underlying KL‐VS action remain to be fully elucidated, the identification of new genetic variants that attenuate AD‐associated changes will bring to light novel molecular targets poised to identify complementary pathways for curbing the disease progression or delaying the onset of symptoms.

### 2.3 CSF collection and analyses

CSF samples (22 mL) were collected in the morning after an 8 to 12 hour fast via lumbar puncture at L3/4 or L4/5 with extraction into polypropylene syringes using a Sprotte 24‐ or 25‐gauge spinal needle, then combined, gently mixed, and centrifuged at 2000 × g for 10 minutes. Supernatants were frozen in 0.5 mL aliquots in polypropylene tubes and stored at −80°C.[Table alz13912-tbl-0001]

Samples were immunoassayed using the NeuroToolKit (NTK; Roche Diagnostics International Ltd) at the Clinical Neurochemistry Laboratory, University of Gothenburg, using the same batch of reagents. The NTK is a panel of robust prototype biomarker assays, used for research purposes only and not approved for clinical use. NTK measures both established (Aβ and tau) and novel AD biomarkers that improve the pathophysiological characterization of AD, including synaptic, axonal, and glial biomarkers.^44^

### 2.4 Statistical analysis

All analyses were done in SPSS, v. 27.0 (IBM). Group differences in demographic characteristics were compared either using one‐way analysis of variance or χ^2^ tests. Covariate‐adjusted (sex, education, *APOE* ε4, family history of AD) regression examined relationships between age group (younger [*N* = 234]; older [*N* = 220]; mean split at age ≥ 62) and the CSF biomarkers of neuroinflammation, synaptic dysfunction, and neurodegeneration. The analyses were then repeated after stratifying the sample by KL‐VS genotype in keeping with the literature^25^, ^26^, ^27^ to determine whether the deleterious effect of age on the CSF markers differed for KL‐VS_HET_ (*N* = 122; younger = 72; older = 50) and non‐carriers (*KL‐VS_NC_*; *N* = 332; younger = 162; older = 170). KL‐VS homozygosity, associated with lower levels of klotho, decreased longevity, and worse cognition, is a rare genotype^17^ and was omitted from analysis due to low sample size (*N* = 5). Significance level with Bonferroni correction for multiple comparisons is set at *P* ≤ 0.006. We also present the results that were significant at the conventional *P* ≤ 0.05, however, to help guide future investigations given the topic is currently grossly lacking in the literature.

## 3 RESULTS

Characteristics of the entire sample, and for KL‐VS_HET_ and *KL‐VS_NC_* separately, are detailed in Table 1. The sample was predominantly White (97%), female (69%) and college educated (16.1 ± 2.42 years) with average age of 61.5 ± 7.75. The Mini‐Mental State Examination scores ranged from 27 to 30, with an average of 29.3 ± 0.96. The sample was enriched for AD risk; 38% were *APOE* ε4 carriers, and 74% had a parental history of AD. Significantly greater percentage of KL‐VS_HET_ compared to of *KL‐VS_NC_* were female (84% vs. 63%) and had a parental history of AD (100% vs. 64%). Approximately 84% to 90% of the sample, depending on the marker, was negative for CSF Aβ and phosphorylated tau (see Table 1) based on our center‐derived cut points.^27^, ^44^

**TABLE 1 alz13912-tbl-0001:** Background characteristics of the total sample and by KL‐VS status.

	Total sample (*N* = 454)	*KL‐VS_NC_* (*N* = 332)	KL‐VS_HET_ (*N* = 122)	*P*
Age (years) M (SD)	61.5 (7.75)	61.99 (7.90)	60.15 (7.19)	0.03
Education (years) M (SD)	16.14 (2.42)	16.15 (2.44)	16.10 (2.39)	0.84
MMSE (total/30) M (SD)	29.27 (2.42)	29.38 (0.70)	29.30 (0.89)	0.25
Female *N* (%)	311 (69)	209 (63)	102 (84)	<0.001
White *N* (%)	438 (97)	318 (96)	120 (98)	0.15
*APOE* ε4 + *N* (%)	170 (38)	122 (37)	48 (39)	0.34
AD parental history *N* (%)	334 (74)	212 (64)	122 (100)	<0.001
^a^Aβ_42/40_‐ *N* (%)	381 (84)	238 (85)	98 (80)	0.13
^a^p‐tau_181_‐ *N* (%)	408 (90)	297 (89)	111 (91)	0.39
^a^p‐tau_181_/Aβ_42_‐ *N* (%)	401 (88)	297 (89)	104 (85)	0.14

^a^ Negative based on our center‐derived cut points for CSF AD biomarkers.^27^, ^44^ ^.^

Abbreviations: AD, Alzheimer's disease; *APOE*, apolipoprotein E; Aβ, amyloid beta; CSF, cerebrospinal fluid; KL‐VS_HET_, *KLOTHO KL‐VS* heterozygotes; *KL‐VS_NC_*, *KLOTHO KL‐VS* non‐carriers; M, mean; MMSE, Mini‐Mental State Examination; p‐tau, phosphorylated tau; SD, standard deviation.

**FIGURE 1:**
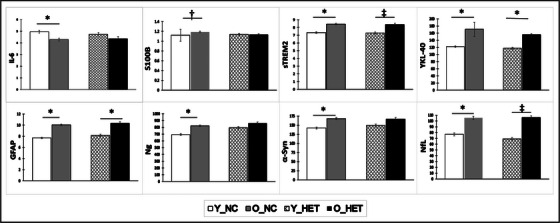
Age‐related differences in CSF analytes of interest across KL‐VS strata. O_NC compared to Y_NC KL‐VS non‐carriers had poorer CSF biomarker profiles across the board, as would be expected (all *P*s ≤ 0.05). This age‐related difference did not reach statistical significance in O_HET for IL‐6 (*P* = 0.43), S100B (*P* = 0.84), Ng (*P* = 0.27), and α‐Syn (*P* = 0.13), and additionally for sTREM2 (*P* = 0.01) and NfL (*P* = 0.01) if the results are considered at Bonferroni adjusted *P* = 0.006. ^*^ *P* ≤ 0.001; ^‡^ *P *= 0.01; ^†^ *P *= 0.05. *P *< 0.006 if Bonferroni adjustment for multiple comparisons is applied. CSF, cerebrospinal fluid; GFAP, glial fibrillary acidic protein; IL‐6, interleukin 6; M, mean; NfL, neurofilament light chain; Ng, neurogranin; O_HET, older KL‐VS heterozygotes; O_NC, older KL‐VS non‐carriers; S100B, S100 calcium‐binding protein B; SE, standard error; sTREM2, soluble triggering receptor expressed on myeloid cells 2; Y_HET, younger KL‐VS heterozygotes; Y_NC, younger KL‐VS non‐carriers; YKL‐40, chitinase‐3‐like protein 1; α‐Syn, total α‐synuclein

None of the CSF biomarkers of interest differed significantly between KL‐VS_HET_ and *KL‐VS_NC_* (all *P*s ≥ 0.44). In the pooled analyses, older age was associated with poorer CSF biomarker profiles across the board (all *P*s < 0.03). In the stratified analyses (Table 2), *KL‐VS_NC_* exhibited this pattern of age‐related differences (all *P*s ≤ 0.001; except S100B [*p* = 0.05] if Bonferroni adjustment [*P* < 0.006] for multiple comparisons is applied), which did not reach statistical significance in KL‐VS_HET_ for S100B (*P* = 0.84), IL‐6 (*P* = 0.43), α‐Syn (*P* = 0.13), and Ng (*P* = 0.27). Additionally, with Bonferroni adjustment, the pattern of age‐related differences does not reach statistical significance in KL‐VS_HET_ for sTREM2 (*P* = 0.01) and NfL (*P* = 0.01). These findings are depicted in Table 2 and Figure 1.

**TABLE 2:** Association between CSF biomarkers and age across KL‐VS strata.

	*KL‐VS_NC_* (*N* = 332)				KL‐VS_HET_ (*N* = 122)					
CSF marker	Age group	B (SE)	M (SE)	F (df)	*P*	B (SE)	M (SE)	F(df)	*P*	% reduction in KL‐VS effect^*^
Markers of inflammation										
IL‐6	<62	0.73 (0.35)	4.93 (3.37)	51.98 (1,327)	<0.001	0.39 (0.5)	4.71 (2.00)	0.62 (1118)	0.43	46.57
	≥62		4.33 (2.99)				4.38 (3.66)			
S100B	<62	−0.06 (0.03)	1.12 (0.26)	3.59 (1327)	0.05^†^	0.01 (0.05)	1.14 (0.30)	0.04 (1118)	0.84	—–
	≥62		1.19 (0.36)				1.13 (0.26)			
sTREM2	<62	−1.17 (0.27)	7.32 (2.09)	16.85 (1327)	<0.001	−1.09 (0.43)	7.28 (2.05)	6.52 (1118)	0.01^‡^, ^#^	6.84
	≥62		8.48 (2.67)				8.41 (2.59)			
YKL‐40	<62	−47.86 (5.83)	121.57 (36.06)	69.36 (1327)	<0.001	−38.32 (6.88)	117.32 (27.54)	31.96 (1118)	<0.001	19.93
	≥62		171.24 (64.23)				156.09 (47.56)			
GFAP	<62	−2.35 (0.32)	7.62 (2.72)	51.98 (1327)	<0.001	−2.20 (0.65)	8.13 (2.97)	11.41 (1118)	0.001	6.38
	≥62		10.1 (3.12)				10.38 (4.15)			
Synaptic Markers										
Ng	<62	−133.38 (33.85)	695.88 (272.16)	14.25 (1327)	<0.001	−62.14 (55.48)	791.07 (288.6)	1.26 (1118)	0.27	53.41
	≥62		820.05(331.69)				861.03 (314.3)			
Markers of neurodegeneration										
α‐Syn	<62	−29.64 (7.36)	141.53 (66.06)	12.28 (1327)	0.001	−17.47 (11.44)	148.69 (61.28)	2.33 (1118)	0.13	41.06
	≥62		168.73 (66.54)				167.52 (61.98)			
NfL	<62	−27.88 (6.92)	75.35 (77.28)	16.91 (1327)	<0.001	−36.80 (6.51)	68.97 (24.49)	6.52 (1118)	0.01^‡^, ^#^	—–
	≥62		106.79 (45.2)				106.49 (46.16)			

*Note*: —– % is not specified when no reduction is present. The percent attenuation in the *KLOTHO KL‐VS* effect among KL‐VS_HET_ compared to *KL‐VS_NC_* ranged from ≈ 7% to 53% (last column). The reduction was determined by direct comparisons of partial regression coefficients (B = unstandardized beta) for each group.

Abbreviations: CSF, cerebrospinal fluid; df, degrees of freedom; GFAP, glial fibrillary acidic protein; IL‐6, interleukin 6; *KL‐VS_HET_, KLOTHO KL‐VS* heterozygotes; *KL‐VS_NC_, KLOTHO KL‐VS* non‐carriers; M, mean; NfL, neurofilament light chain; Ng, neurogranin; S100B, S100 calcium‐binding protein B; SE, standard error; sTREM2, soluble triggering receptor expressed on myeloid cells 2; YKL‐40, chitinase‐3‐like protein 1; α‐Syn, total α‐synuclein.

* Significant at *P* ≤ 0.001.

^‡^ Significant at *P* = 0.01.

^†^ Significant at *P* = 0.05.

^#^ *P* < 0.006 if Bonferroni adjustment for multiple comparisons is applied.

**FIGURE 2:**
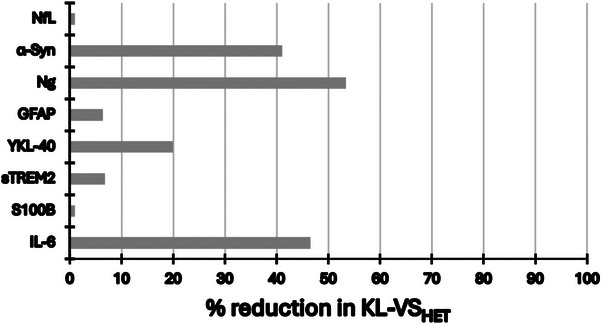
The percent attenuation in the age effect among KL‐VS_HET_ compared to *KL‐VS_NC_*. Percent reduction in age effect was obtained via direct comparison of β values for each group and ranged from 6% to 53%. Even when significant differences between younger and older groups persisted regardless of genotype, the effects within the *KL‐VS_NC_* were markedly stronger compared to KL‐VS_HET_ (GFAP [≈ 6%], sTREM2 [7%] and YKL‐40 [≈ 20%]). GFAP, glial fibrillary acidic protein; KL‐VS_HET_, *KLOTHO KL‐VS* heterozygotes; *KL‐VS_NC_*, *KLOTHO KL‐VS* non‐carriers; sTREM2, soluble triggering receptor expressed on myeloid cells 2; YKL‐40, chitinase‐3‐like protein 1

The percent attenuation in the age effect among KL‐VS_HET_ compared to *KL‐VS_NC_*, obtained via direct comparison of β values for each group, ranged from 6% to 53% (Table 2, last column; Figure 2). Although significant differences between younger and older groups were evident in both *KL‐VS_NC_* (*P*s ≤ 0.001) and KL‐VS_HET_ (*P*s ≤ 0.01), the effects within the non‐carriers were markedly stronger compared to heterozygotes for GFAP (≈ 6%), sTREM2 (≈ 7%), and YKL‐40 (≈ 20%).

Because the sample size of *KL‐VS_NC_* was nearly three times that of KL‐VS_HET_ (332 vs. 122) we repeated the foregoing analyses on a subsample of 122 *KL‐VS_NC_* who were matched on sex and age to the 122 KL‐VS_HET_ to rule out the possibility that our results were due to sample size differences. A nearly identical pattern of results was observed in the matched subsample analyses compared to that observed with the full sample. Specifically, older compared to younger *KL‐VS_NC_* had greater S100B (*P* = 0.01), sTREM2 (*P* = 0.03), and YKL‐40 (*P* = 0.01) and NFL (*P =* 0.02). The age‐related differences were not significant in KL‐VS_HET_ (all *P*s > 0.2).

## 4 DISCUSSION

Our findings suggest that the functionally advantageous KL‐VS_HET_ variant seems to protect against age‐related biomolecular alterations that confer risk for AD dementia. Specifically, in this late‐middle‐aged and older adult cohort enriched for AD risk, the expected unfavorable age‐related changes in neuroinflammation (IL‐6 and S100B), synaptic dysfunction (Ng), and neurodegeneration (α‐Syn) were attenuated in KL‐VS_HET_.

IL‐6 is an multifunctional cytokine that can be pro‐ or anti‐inflammatory depending on the signaling pathway and the amount released.^45^ Removal of IL‐6 results in reduced microglial activation and overproduction leads to chronic neuroinflammation and neurodegeneration.^46^ IL‐6 is elevated in both the CSF and serum of people with AD^47^, ^48^ and higher peripheral levels in late midlife predict cognitive decline some 10 years later.^49^ Although one might expect the levels of IL‐6 to rise with age, in our sample we see lower average IL‐6 levels in older adults. This observation, albeit counterintuitive, is shared with others^50^ and it has been since suggested that high IL‐6 production is not a consequence of the normal aging process and, if observed, likely represents an undiagnosed underlying condition.^51^ Neuroinflammation and neurodegeneration are a part of a vicious cycle of Aβ deposition, neuronal injury, tangle formation, and neuronal death in AD. Sustained inflammation in AD brains, originally thought to be reactive to the neuronal loss associated with the disorder, facilitates and exacerbates both Aβ and tau pathologies and may even provide a link between the initial Aβ pathology and the later development of tangles, and as such, has been suggested as a mechanism potentially central to the accumulation and progression of AD neuropathology.^52^

S100B is predominantly an astrocytic protein with cytokine‐like functions and is considered a putative marker of glial injury.^53^, ^54^, ^55^ It is hypothesized that S100B contributes to disease progression^53^, ^56^ via microglia‐induced neurodegeneration by causing synaptic dysfunction and by direct phagocytosis of neurons,^57^ given the cross‐talk between microglia and astrocytes whereby activation of one stimulates the release of cytokines and chemokines that in turn activate the other.^58^, ^59^, ^60^ S100B is significantly elevated in the CSF of AD patients compared to controls^61^ and correlates with brain atrophy.^62^

Ng, a calmodulin‐binding postsynaptic protein expressed predominantly in postsynaptic spines of the dendrites, is involved in postsynaptic signaling pathways including memory processes.^63^ Ng levels are higher in AD patients compared to controls^64^, ^65^, ^66^, ^67^ and can predict with > 90% sensitivity and specificity^68^ who converts to AD from mild cognitive impairment (MCI).^69^ Ng concentration also differentiates early AD patients from controls with diagnostic utility comparable to core AD CSF biomarkers,^70^, ^71^ and increases over time in cognitively unimpaired but not MCI or AD patients suggesting that Ng is tracking with synaptic dysfunction or loss during pre‐symptomatic stages.^69^

Accumulating evidence suggests that the presynaptic protein α‐Syn is also involved in the pathophysiology of AD.^72^ Abnormal forms of α‐Syn are thought to trigger progressive neurodegeneration via mitochondrial impairment, lysosomal dysfunction and altered calcium homeostasis, plasticity, and synaptic function preceding neurodegeneration.^73^ Furthermore, evidence from experimental models suggests that the presence of inflammation precedes deposition and spread of α‐Syn and thereby linking inflammation and synaptic dysfunction.^73^ Higher CSF α‐Syn has been linked to cognitive decline in MCI and AD patients,^74^ and to accumulation of Aβ plaques in both asymptomatic individuals at risk for AD and individuals carrying autosomal dominant AD mutations.^72^ Higher α‐Syn levels have also been linked to tau hyperphosphorylation and pathological actions of both Aβ and the ε4 allele of the *APOE* gene.^72^

In summary, we report that KL‐VS_HET_ attenuates deleterious effects of age on CSF IL‐6, S100B, Ng, and α‐Syn in light of the growing literature suggesting a vicious cycle between inflammatory processes, synaptic dysfunction, and neurodegeneration and their known contributions to AD pathology. The attenuation in KL‐VS_HET_, albeit observed across all CSF biomarkers of interest regardless of statistical significance, was also the strongest for IL‐6, S100B, Ng, and α‐Syn. KL‐VS_HET_ hence appears to protect against an array of deleterious age‐related biomolecular changes related to this complex disease in addition to the previously reported attenuation of core AD biomarkers,^25^, ^26^, ^27^ which is important given the multifactorial nature of AD.

The study is not without limitations. Our cohort is predominantly White and highly educated. Moreover, the sample is enriched for parental history of AD and consequently many are *APOE* ε4 allele carriers, resulting in higher prevalence of both exposures than what is normally observed in the general population. These sample characteristics may potentially limit the generalizability of our findings. Last, the cross‐sectional nature of the present study may be seen as a limitation. This, however, is addressable in the future as both WRAP and WADRC are prospective cohorts and data collection is ongoing.

While the mechanism underlying KL‐VS_HET_ action remains to be fully elucidated, the identification of new genetic variants that positively modify AD risk, such as *KLOTHO*, will bring to light novel molecular targets for future therapeutic trials. This line of research is poised to identify complementary pathways for curbing the disease progression and delaying the onset of symptoms.

## CONFLICT OF INTEREST STATEMENT

All authors report no conflict of interest directly related to this study. S.C.J. has served on advisory boards for ALZPath and Enigma Biosciences. K.B. has served as a consultant, on advisory boards, or on data‐monitoring committees for Acumen, ALZPath, AriBio, BioArctic, Biogen, Eisai, Lilly, Moleac Pte. Ltd, Novartis, Ono Pharma, Prothena, Roche Diagnostics, and Siemens Healthineers; has served on data monitoring committees for Julius Clinical and Novartis; has given lectures, produced educational materials, and participated in educational programs for AC Immune, Biogen, Celdara Medical, Eisai, and Roche Diagnostics; and is a co‐founder of Brain Biomarker Solutions in Gothenburg AB, which is a part of the GU Ventures Incubator Program (outside submitted work). H.Z. has served on scientific advisory boards and/or as a consultant for Abbvie, Acumen, Alector, Alzinova, ALZPath, Annexon, Apellis, Artery Therapeutics, AZTherapies, Cognito Therapeutics, CogRx, Denali, Eisai, Merry Life, Nervgen, Novo Nordisk, Optoceutics, Passage Bio, Pinteon Therapeutics, Prothena, Red Abbey Labs, reMYND, Roche, Samumed, Siemens Healthineers, Triplet Therapeutics, and Wave; has given lectures in symposia sponsored by Alzecure, Biogen, Cellectricon, Fujirebio, Lilly, and Roche; and is a co‐founder of Brain Biomarker Solutions in Gothenburg AB, which is a part of the GU Ventures Incubator Program (outside submitted work). B.B.B. has received precursor and reagents from AVID Radiopharmaceuticals and has served as an advisor to Cognito Therapeutics, Merry Life, and New Amsterdam. G.K. is a full‐time employee of Roche Diagnostics GmbH. C.Q.‐R. is a full‐time employee of Roche Diagnostics International Ltd. All other authors have no disclosures to report. KLOTHO is the subject of a pending international patent application held by the Regents of the University of California. The NeuroToolKit is a panel of exploratory prototype assays designed to robustly evaluate biomarkers associated with key pathologic events characteristic of AD and other neurological disorders, used for research purposes only and not approved for clinical use (Roche Diagnostics International Ltd). Elecsys Aβ(1–42) CSF and Elecsys Phospho‐Tau (181P) CSF assays are approved for clinical use. All other product names and trademarks are the property of their respective owners. Author disclosures are available in the supporting information.

## CONSENT STATEMENT

Written informed consent was provided by all participants prior to study participation and approval was obtained from the institutional review board.

## Supporting information

Supporting Information
